# Sea buckthorn and its flavonoids isorhamnetin, quercetin, and kaempferol favorably influence bone and breast tissue health

**DOI:** 10.3389/fphar.2024.1462823

**Published:** 2024-10-09

**Authors:** Monika Martiniakova, Noemi Penzes, Roman Biro, Anna Sarocka, Veronika Kovacova, Vladimira Mondockova, Sona Ciernikova, Radoslav Omelka

**Affiliations:** ^1^ Department of Zoology and Anthropology, Faculty of Natural Sciences and Informatics, Constantine the Philosopher University in Nitra, Nitra, Slovakia; ^2^ Department of Botany and Genetics, Faculty of Natural Sciences and Informatics, Constantine the Philosopher University in Nitra, Nitra, Slovakia; ^3^ Department of Genetics, Cancer Research Institute, Biomedical Research Center of Slovak Academy of Sciences, Bratislava, Slovakia

**Keywords:** sea buckthorn, isorhamnetin, quercetin, kaempferol, bone tissue, breast tissue, osteoporosis, breast cancer

## Abstract

Bone tissue and breast tissue are interrelated, as demonstrated by breast microcalcifications, breast cancer bone metastases, bone morphogenetic proteins, and Wnt signaling. In addition, osteoblasts and osteoclasts represent an important switch of tumor cell dormancy during bone metastasis. Damage to both types of tissues mentioned above is highly prevalent, especially in postmenopausal women, and manifests itself in osteoporosis and breast cancer. Sea buckthorn (*Elaeagnus rhamnoides* L.), a botanical drug with high antioxidant, antitumor, anti-inflammatory, immunomodulatory, and regenerative properties, has great therapeutic potential due to the unique composition of its bioactive metabolites. This review aimed to summarize the current knowledge from *in vitro* and *in vivo* studies on the effect of sea buckthorn, as well as its most widespread flavonoids isorhamnetin, quercetin, and kaempferol, on bone and breast tissue health. *In vitro* studies have revealed the beneficial impacts of sea buckthorn and aforementioned flavonoids on both bone health (bone remodeling, mineralization, and oxidative stress) and breast tissue health (cancer cell proliferation, apoptosis, tumor growth, and metastatic behavior). *In vivo* studies have documented their protective effects against disturbed bone microarchitecture and reduced bone strength in animal models of osteoporosis, as well as against tumor expansion and metastatic properties in animal xenograft models. In any case, further research and clinical trials are needed to carefully evaluate the potential therapeutic benefits of sea buckthorn and its flavonoids. Based on the available information, however, it can be concluded that these bioactive metabolites favorably affect both bone and breast tissue health.

## 1 Introduction

Sea buckthorn (*Elaeagnus rhamnoides* L.) is a nitrogen-fixing thorny deciduous shrub that is naturally distributed in Asia and Europe. It can grow in difficult conditions such as frost, drought, and polluted air. This botanical drug has been widely used for its nutritional and medicinal purposes (Patel et al., 2012; Jaśniewska and Diowksz, 2021). Raw fruits, various products made from them (e.g., juices, jams, tinctures), alcoholic extracts from different parts of this botanical drug and oil from the seeds have shown to possess antioxidant, antitumor, anti-inflammatory, immunomodulatory, and regenerative properties, which are related to a unique composition of bioactive metabolites, rich in phenolic metabolites (mainly phenolic acids and flavonoids), essential fatty acids (palmitic acid, palmitoleic acid, stearic acids), vitamins (A, B, C, E, K), phytosterols (cycloartenol, campesterol, sitosterol), and carotenoids (lycopene, lutein, zeaxantin) (Olas et al., 2018; Dudau et al., 2021; Stochmal et al., 2022). In general, more than 60 flavonoids and 10 phenolic acids have been identified in sea buckthorn. The most abundant flavonoids in fruits, leaves, and seeds are isorhamnetin and quercetin, followed by kaempferol, luteolin, myricetin, syringetin, naringenin, and epicatechin (Ren et al., 2020; He et al., 2023). Considering phenolic acids, gallic acid, caffeic acid, and ferulic acid are present in leaves and fruits (Jaśniewska and Diowksz, 2021; Danielski and Shahidi, 2024). This broad spectrum of bioactive metabolites can help prevent or treat a variety of conditions, such as cardiovascular diseases, *diabetes mellitus*, liver damage, gastrointestinal disorders, neuronal damage, skin lesions, retina damage, osteoporosis, and tumors (Ren et al., 2020).

In this review, we summarize the current knowledge from *in vitro* and *in vivo* studies regarding the effect of sea buckthorn, as well as its most widespread flavonoids isorhamnetin, quercetin, and kaempferol, on bone and breast tissue health. In individual studies, where it was relevant, we assessed the fulfillment of the requirements for the phytochemical characterization of the analysed extracts according to the ConPhyMP (Heinrich et al., 2022). Selected flavonoids exhibit both protective effects on bone tissue and antitumor impacts on breast tissue, potentially ameliorating bone loss in osteoporosis and inhibiting breast cancer progression. Therefore, we hypothesized that all of these bioactive metabolites should simultaneously attenuate bone damage and breast malignancy. These profitable effects of aforementioned bioactive metabolites on the health status of bone and breast tissues are still not sufficiently presented, as well as the interplay between both tissues.

## 2 The link between bone tissue and breast tissue

**FIGURE 1:**
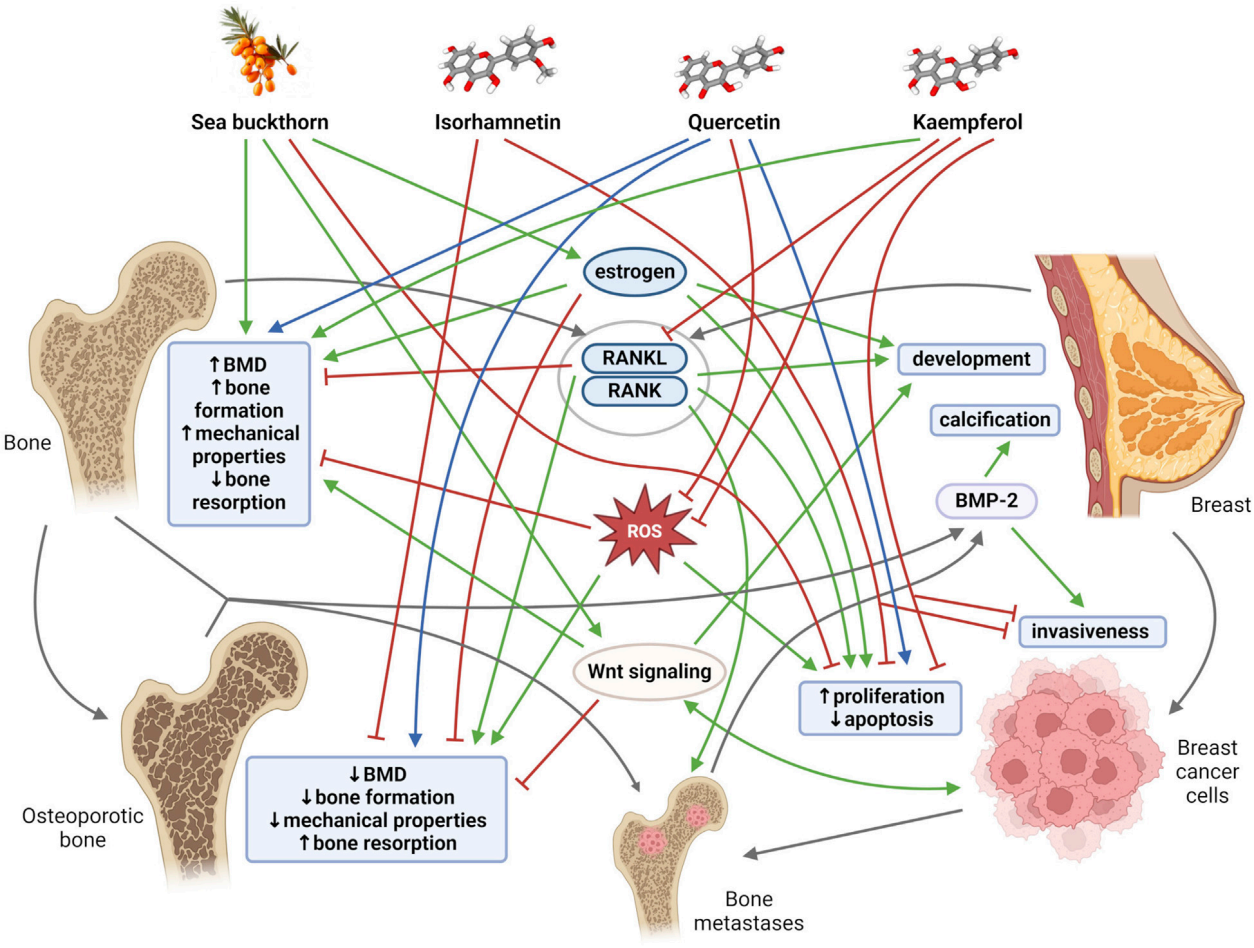
General mechanisms and links among bone tissue, breast tissue, sea buckthorn, its flavonoids (isorhamnetin, quercetin, kaempferol). Blunt red arrows indicate an inhibitory effect, sharp green arrows designate a stimulatory effect. Blue arrows show a dose-different effect. Gray arrows indicate the connection of individual mechanisms, molecules and structures. BMD, bone mineral density; BMP-2, bone morphogenetic protein-2; RANK, receptor activator of nuclear factor kappa β; RANKL, receptor activator of nuclear factor kappa β ligand; ROS, reactive oxygen species. Created with BioRender.com using chemical structures from PubChem (Kim et al., 2023); CIDs: 5281654, 5280343, 5280863, 122228.

The relationships between bone and breast tissues are generally based on a constant dependence on the same regulatory and signaling molecules, as well as on mutual interactions through tissue-specific molecules that are mainly exposed at specific periods of life. Both bone tissue and breast tissue are dependent on estrogen, a key hormone that regulates bone mineral density (BMD), thereby maintaining the balance between bone formation and bone resorption. Estrogen is also an important mediator of mammary gland morphogenesis (Stingl, 2011). Furthermore, receptor activator of nuclear factor kappa β (RANK) and its ligand RANKL were discovered as key regulators of osteoclast development and activation. RANK and RANKL, however, also play an important role in the development of a functional lactating mammary gland during pregnancy (Sigl and Penninger, 2014). The mammary gland and bones are closely linked during lactation, when increased calcium requirements for milk production change bone and mineral metabolism (Athonvarangkul and Wysolmerski, 2023). In addition, with increasing age, cells and tissues, including breast and bone, become more susceptible to oxidative stress, which could modify the activity of key proteins and pathways needed to protect against bone and breast tissue damage (Figure 1). Disruption of the aforementioned important regulatory mechanisms and age-related changes in the organism, including oxidative stress, inflammation, and lipid accumulation contribute to simultaneous occurrence of osteoporosis and breast cancer in postmenopausal women (Cho et al., 2015; Muhammad et al., 2018; Martiniakova et al., 2023a). Overall, osteoporosis is the most common type of bone disease and its prevalence was reported at 18.3%. By 2050, more than 30 million people in Europe are expected to be affected by osteoporosis (Salari et al., 2021; Martiniakova et al., 2024). Globally, breast cancer is the second most common cause of cancer death in women, representing 12.5% of all new annual cancer cases worldwide (Muhammad et al., 2018; World Health Organization International Agency for Research on Cancer, 2024). There is a close clinically significant relationship between osteoporosis and breast cancer. Estrogen deficiency is considered the main cause of postmenopausal osteoporosis. Conversely, elevated estrogen levels during life (e.g., late menopause, obesity, estrogen replacement therapy) are associated with increased incidence of breast malignancy. Deregulation of the RANK/RANKL system also contributes to the development of postmenopausal osteoporosis. The RANK/RANKL pathway has also been found to be involved in hormone-induced breast cancer development and metastatic spread to bone (Muhammad et al., 2018; Martiniakova et al., 2023a).

Another clinical interrelationship between bone tissue and breast tissue is demonstrated by breast microcalcifications and breast cancer bone metastases (Antonacci et al., 2018). The presence of breast microcalcifications and localized deposits of hydroxyapatite in the breast tissue is actually considered an early mammographic sign of breast cancer (Johnson et al., 1999). Approximately 70%–80% of patients with advanced breast cancer experienced bone metastases (Chen et al., 2010), which seriously affect their quality of life and can lead to death. Moreover, osteoblasts and osteoclasts represent an important switch in tumor cell dormancy during bone metastasis (Haider et al., 2020; Dai et al., 2022). Several researchers have also demonstrated the expression of typical bone markers in breast cells, including predominantly bone morphogenetic proteins (BMPs) and Wnt signaling (Liu et al., 2008; Bramwell et al., 2014). In general, BMPs are cytokines belonging to the transforming growth factor (TGF)-β superfamily that play multiple functions during development and tissue homeostasis, including the regulation of bone homeostasis (Sánchez-Duffhues et al., 2015). Current studies have shown that BMPs may also be involved in breast tissue. They can support oncogenic behavior by affecting apoptosis, migration, invasion, and angiogenesis (Alarmo and Kallioniemi, 2010), initiate microcalcification (Liu et al., 2008), and promote the phenomenon of epithelial-mesenchymal transition (EMT) (Gonzalez and Medici, 2014). Canonical Wnt signaling activity supports bone formation (Turashvili et al., 2006), and it is also involved in several stages of mammary gland growth and differentiation and human breast cancer development (Howe and Brown, 2004). Additionally, it is also important to mention that the implication of other bone-derived factors (e.g., osteocalcin, fibroblast growth factor 23, sclerostin, lipocalin 2) in cancer biology has attracted research interest in recent years (Mansinho et al., 2019; Santiago-Sánchez et al., 2020; Martiniakova et al., 2023b), as they may be used as promising tumor biomarkers. Conversely, breast cancer cells are able to secrete various cytokines, including parathyroid hormone (PTH)-related protein, vascular endothelial growth factor (VEGF), RANKL, various interleukins (ILs) that serve as crucial bone modulators (Shemanko et al., 2016). These facts also contribute to a profound interplay between bone tissue and breast tissue.

Current pharmacological treatments for both osteoporosis and breast cancer often cause undesirable side effects; therefore, various natural metabolites, including those found in sea buckthorn, are being intensively researched to discover an alternative and effective treatment method with less harmful impacts (Li Y. et al., 2017; Martiniakova et al., 2020).

## 3 The impact of sea buckthorn and its flavonoids isorhamnetin, quercetin, and kaempferol on bone tissue health

Several studies have shown that sea buckthorn fatty acids significantly elevated levels of serum estrogen, TGF, insulin-like growth factor (IGF), BMD, cortical bone thickness, trabecular number, and bone mechanical properties in aged female rats (Liu et al., 2006b; Liu et al., 2006a). In this context, Yuan et al. (2022) found that sea buckthorn was able not only to increase BMD and estrogen levels, but also to raise levels of bone turnover markers (e.g., procollagen type 1N propeptide: P1NP, C-terminal telopeptide: CTX) and improve trabecular bone microstructure (relative bone volume, trabecular thickness) in ovariectomized (OVX) rats. Furthermore, it enhanced the efficacy of a traditional Chinese medicine QiangGuYin (used to treat osteoporosis) by inhibiting Casein kinase 2-interacting protein-1 (CKIP-1) and Notum expression through the Wnt/β-catenin pathway. Molecular docking analysis revealed that seven active components, including isorhamnetin, quercetin, and kaempferol, were able to potentially influence CKIP-1 and Notum (Yuan et al., 2022). However, this study did not provide important details about the material used (Supplementary Table 1). Moreover, the discovery of the ‘active’ metabolites of sea buckthorn was based only on the calculated results of molecular docking and was not followed by experimental studies. According to Park et al. (2022), an alcoholic extracts of sea buckthorn fruits and their fractions increased BMD and exerted the protective effects against cartilage damage and disturbed trabecular bone microarchitecture. Moreover, the extracts and their fractions stimulated the differentiation of murine mesenchymal stem cells into osteoblasts and elevated gene expression of osteogenic factors and markers, e.g., alkaline phosphatase (ALP), runt-related transcription factor 2 (RUNX2), osteopontin (OPN), osterix (OSX). Lee et al. (2023) pointed out the anti-osteoporotic impacts of triterpenoids from sea buckthorn fruit by promoting osteoblast differentiation from mesenchymal stem cells. However, the extracts used in the studies mentioned above would require better characterization. Relevant information related to this issue is presented in Supplementary Table 1; Figure 1.

There is a limited number of studies examining the effect of isorhamnetin in relation to bone damage. According to Yamaguchi et al. (2007), isorhamnetin inhibited PTH-stimulated osteoclastogenesis in mouse bone marrow cells and elevated PTH-decreased calcium content in femoral-diaphyseal tissue cultures. Moreover, isorhamnetin reduced osteoclast formation in bone marrow macrophages through inhibition of mitogen-activated protein kinase (MAPK), nuclear factor-kappa B (NF-κB), and protein kinase B (AKT) signaling (Zhou et al., 2019).

In contrast to isorhamnetin, more *in vitro* studies have shown the ability of quercetin to reduce osteoblast apoptosis, osteoclastogenesis, and oxidative stress (Woo et al., 2004; Yamaguchi et al., 2007; Tsuji et al., 2009; Yamaguchi and Weitzmann, 2011; Tripathi et al., 2015; Niu et al., 2020; Wong et al., 2020). On the contrary, quercetin significantly increased osteoblastogenesis, ALP activity, calcium content, expression of bone formation-associated proteins in mouse preosteoblastic MC3T3-E1 cells (Tripathi et al., 2015), rat osteoblast-like ROS 17/2.8 cells (Kim et al., 2007), human osteoblast-like MG-63 cells (Prouillet et al., 2004), and bone marrow mesenchymal stem cells (BMSCs) (Yuan et al., 2018; Feng et al., 2023). In some researches mentioned above, the effect of quercetin on NF-κB and Wnt/β-catenin signaling was also reported. Notably, higher doses of quercetin had either suppressive or decreased activity on osteoblast-specific gene expression, osteoblast growth and mineralization in several studies (Yamaguchi and Weitzmann, 2011; Wong et al., 2020). Furthermore, quercetin has an ability to bind to the estrogen receptor (ER) (Ross and Kasum, 2002) and influence osteoblast and osteoclast activity, as well as the expression and activity of various inflammatory cytokines, participating in bone remodeling (Wang et al., 2017). Numerous *in vivo* studies have established the protective effects of quercetin against bone loss through increased BMD, improved bone microarchitecture and bone strength, elevated bone growth, decreased bone resorption markers, and increased bone formation markers (Tsuji et al., 2009; Oršolić et al., 2018; Yuan et al., 2018; Niu et al., 2020; Sun et al., 2022; Feng et al., 2023). Yurteri et al. (2023) revealed that quercetin was also able to strengthen bone in both the early and late stages of fracture healing.

Several *in vitro* studies have shown that inhibitory effects of kaempferol on osteoclastogenesis may be linked to the downregulation of osteoclastogenic factors, such as RANKL, nuclear factor of activated T-cells cytoplasmic 1 (NFAT-c1), tumor necrosis factor receptor-associated factor 6 (TRAF6), c-Fos proto-oncogene, NF-κB signaling (Pang et al., 2006; Lee et al., 2014; Kim et al., 2018; Wong et al., 2019). On the other hand, conducted experiments have reported that kaempferol increased ALP activity and promoted osteogenic differentiation and mineralization in rat bone marrow mesenchymal stem cells (rBMSCs) via mediation of SOX2/miR-124-3p/PI3K/AKT/mTOR axis as well as in osteoblastic MC3T3-E1 cells by inducing autophagy and activating osteoblast differentiation markers such as RUNX2, OSX, collagen 1 (Kim I. R. et al., 2016; Xie et al., 2021; Gan et al., 2022). In addition, kaempferol could affect bone through the regulation of AKT serine/threonine kinase 1 (*AKT1*) and matrix metalloproteinase (*MMP*)*-9* gene expressions, which are closely related to the pathogenesis of bone loss (Dong et al., 2024). Furthermore, kaempferol can modulate bone metabolism also through the ER (Jia et al., 2012). Tang et al. (2008) revealed that kaempferol activated ERβ-mediated estrogen response element (ERE) transcription in MG-63 cell line. *In vivo* studies have demonstrated that kaempferol can increase BMD and ALP activity, reduce bone turnover, and improve trabecular bone microarchitecture and bone strength in OVX rats (Trivedi et al., 2008; Nowak et al., 2017; Liu et al., 2021). Moreover, kaempferol was found to be able to ameliorate osteoporosis by raising C-X-C motif ligand 12 (CXCL12) expression and decreasing miR-10a-3p (Liu et al., 2021).

## 4 The impact of sea buckthorn and its flavonoids isorhamnetin, quercetin, and kaempferol on breast tissue health

Several *in vitro* studies have demonstrated the antitumor activity of sea buckthorn. Zhang et al. (2005) examined changes in apoptosis-related gene expression profiles in human breast carcinoma Bcap-37 cells induced by flavonoids from sea buckthorn seeds. These authors found that the expression of 32 apoptosis-related genes (e.g., *IGFBP4*, *CTNNB1*, *CASP3*, *GADD34*) was affected by flavonoid treatment. According to Wang et al. (2014), sea buckthorn procyanidins isolated from the seeds could induce apoptosis of human breast cancer MDA-MB-231 cells through fatty acid synthase inhibition. Indeed, high levels of this enzyme have been identified in cancer cells. Olsson et al. (2004) reported that sea buckthorn fruit extracts reduced the proliferation of breast cancer MCF-7 cells in a concentration-dependent manner. Similarly, Boivin et al. (2007) determined the inhibition of breast cancer (MCF-7 and MDA-MB-231) cell proliferation by sea buckthorn berry juice, as well as the suppression of tumor necrosis factor (TNF)-induced activation of the nuclear transcription factor NF-κB. A limitation of the studies mentioned above is the lack of characterization of the experimental material. In addition, many studies have determined a positive role of sea buckthorn oil in cancer treatment, including chemotherapy and radiotherapy (Olas, 2018; Olas et al., 2018). Table 1; Figure 1 summarize relevant information related to this issue.

**TABLE 1:** *In vitro* and *in vivo* studies reflecting the potential of sea buckthorn and its flavonoids isorhamnetin, quercetin, and kaempferol against breast cancer.

Research model	Applied treatment and metabolite description	Obtained results	References
*In vitro:* cells MDA-MB-231 overexpressed fatty acid synthase Control: negative	Dried procyanidins isolated from sea buckhorn seeds; 0–60 μg/mL/24 h Herbal parts: dried powder seeds Minimal active concentration: 0.087 μg/mL Extraction details: solvent (70% ethanol, anhydrous ethanol, 50% ethanol), type (liquid), drug to solvent ratio 1:10, drug to extract ratio 100:1 Methods for procyanidin determination: ammonium ferric sulfate assay No data about authentication of the plant material, its origin, locality and date of harvesting, deposition of voucher specimen. The extract is poorly characterized by analytical methods	↓ Fatty acid synthase activity ↓ Viability of the cells ↑ Apoptosis of the cells	Wang et al. (2014)
*In vitro:* cells MCF-7 Control: negative	Extracts of sea buckhorn 0.025, 0.05, 0.25, and 0.5%/24 h Herbal parts: fruits Origin: university assortment, collected from wild populations Minimal active concentration: 0.025% Extraction details: solvent (ethanol/water, H_3_PO_4_), type (liquid) Methods for extract characterization: HPLC-UV, HPLC-DAD No data about authentication of the plant material	↓ Proliferation of the cells	Olsson et al. (2004)
*In vitro:* cells MCF-7, MDA-MB-231 Control: negative	Sea buckhorn berry juice; 10, 20, 30, 40, and 50 μL/mL/48 h (proliferation) 25 μL/mL/24 h (viability) Herbal parts: berries Authentication: cultivar Sunny Origin: obtained from local farmers Extraction details: prepared by a centrifugal extractor No data about authentication of the plant material, its exact origin, locality and date of harvesting, deposition of voucher specimen. The extract is not characterized by analytical methods	↓ Proliferation of the cells ↓ Expression of cdk4, cdk6, cyclin D1 and cyclin D3 ↓ TNF-induced activation of the nuclear transcription factor NFκB	Boivin et al. (2007)
*In vitro:* cells MCF-7, T47D, BT474, BT549, MDA-MB-231, MDA-MB-468 Control: positive and negative	Isorhamnetin; 0.4, 1.2, 3.7, 10, 11.1, 30, 33.3, 100 μM/48 h Manufacturer and/or supplier of the product: Shanghai Tongtian Biotechnology Co., Ltd., Shanghai, China Product name: Isorhamnetin Minimal active concentration: 10 μM	↓ Proliferation of cancer cells ↑ Apoptosis of cancer cells ↓ AKT/mTOR and MAPK/ERK signaling pathways	Hu et al. (2015)
*In vitro:* cells MDA-MB-231 Control: negative	Isorhamnetin; 10, 20, or 40 μM/24 h Manufacturer and/or supplier of the product: Sichuan Weikeqi Biological Technology co., ttd., Sichuan, China Product name: Isorhamnetin Minimal active concentration: 20 μM	↓ Viability of the cells ↓ Adhesion, migration, and invasion of the cells ↓ Activity and expression of MMP-2 and MMP-9 ↓ p38 MAPK and STAT3	Li et al. (2015a)
*In vitro:* cells MCF-7 Control: positive and negative	Isorhamnetin; 25, 50 and 100 μM/48 h Manufacturer and/or supplier of the product: synthesized by Paul W. Needs Product name: Isorhamnetin Minimal active concentration: 25 μM	↓ Growth of the cells ↑ Apoptosis of the cells ↑ Cytotoxicity	Wu et al. (2018a)
*In vitro:* cells MCF-7/ADR, MDA-MB-231/DOX (both doxorubicin-resistant) Control: positive and negative	Isorhamnetin; 10, 20, 30, 50 μM/24 or 48 h Manufacturer and/or supplier of the product: Shanghai Tongtian Biotechnology Co., Ltd., Shanghai, China Product name: Isorhamnetin Minimal active concentration: 10 μM	↓ Proliferation and migration of drug-resistant cells ↑ Cell cycle arrest and apoptosis ↑ Intracellular ROS and DNA damage ↓ mTOR pathway	Yang et al. (2023)
*In vitro:* cells MCF-7 Control: positive and negative	Quercetin; 25, 50 and 100 μM/48 h Manufacturer and/or supplier of the product: National Institute for the Control of Pharmaceutical and Biological Products, Beijing, China Product name: Quercetin Minimal active concentration: 25 μM	↓ Growth of the cells ↑ Apoptosis of the cells ↑ Cytotoxicity	Wu et al. (2018a)
*In vitro:* cells MCF-7 Control: negative	Quercetin; 2.5, 5, 10, 20 and 40 mg/mL/24 or 48 h Product name: Quercetin Manufacturer and/or supplier of the product: Sigma, St. Louis, MO, United States Minimal active concentration: 40 mg/mL	↓ Proliferation and growth of the cells ↑ Apoptosis of the cells	Deng et al. (2013)
*In vitro:* Cells MDA-MB-231 Control: negative	Quercetin; 50, 100, 150, 200, 250, and 300 μM/12 and 24 h Manufacturer and/or supplier of the product: Sigma, St. Louis, MO, United States Product name: Quercetin Minimal active concentration: 100 μM	↓ Viability of the cells ↑ Apoptosis (higher concentrations) ↑ Caspase-3, -8 and -9, bax ↑ DNA damage	Chien et al. (2009)
*In vitro:* cells MCF-7 Control: negative	Quercetin; 10–175 μM/24 and 48 h Manufacturer and/or supplier of the product: Sigma, St. Louis, MO, United States Product name: Quercetin Minimal active concentration: 10 μM	↓ Viability of the cells ↑ Number of S phase and sub-G1 phase cells ↓ CDK2, cyclins A and B ↑ p53 and p57, caspase-6, -8 and -9	Chou et al. (2010)
*In vitro:* cells MCF-7, MDA-MB-231 Control: positive and negative	Quercetin; 0.1–500 μM/12–96 h Manufacturer and/or supplier of the product: Sigma, St. Louis, MO, United States Product name: Quercetin Minimal active concentration: 5 μM	↑ Cell growth of MCF-7 at low concentrations (1–20 μM) ↓ Cell viability at higher concentrations (≥50 μM) in both cell lines	Xu et al. (2020)
*In vitro:* cells MCF-7 and T47D (ER-positive), HCC-38 and MDA-MB-231 (ER- negative) Control: negative	Quercetin; 0–100 μM/24 h Manufacturer and/or supplier of the product: Acros Organics, Nj, United States Product name: Quercetin Minimal active concentration: 10 μM	↓ Proliferation of ER- negative cells ↑ Proliferation of ER-positive cells at lower concentrations ↓ Proliferation of ER-positive cells at concentrations higher than 45 or 55 μM for T47D and MCF-7, respectively	van der Woude et al. (2003), van der Woude et al. (2005)
*In vitro:* cells MCF-7 Control: negative	Quercetin; 12.5, 25, 50, 100, 200 μM/24, 48 and 72 h Manufacturer and/or supplier of the product: Sigma, St. Louis, MO, United States Product name: Quercetin Minimal active concentration: 12.5 μM	↓ Proliferation of the cells (>50 μM) ↑ Apoptosis of the cells (>50 μM)	Duo et al. (2012)
*In vitro:* cells MCF-7, MDA-MB-231; *In vivo:* xenograft mouse model (n = 6 per group) Control: negative	Quercetin; 25, 50, 80, 100 μM/48 h Manufacturer and/or supplier of the product: not provided Product name: Quercetin Minimal active concentration: 25 μM	↓ Proliferation of both cell types by miR-146a upregulation ↑ Apoptosis of both cell types through caspase-3 activation ↓ Tumor invasion through EGFR downregulation	Tao et al. (2015)
*In vitro:* cells MCF-7 Control: positive and negative	Quercetin; 25, 50 and 100 μM/48 h Manufacturer and/or supplier of the product: National Institute for the Control of Pharmaceutical and Biological Products, Beijing, China Product name: Quercetin Minimal active concentration: 25 μM	↓ Cell growth ↑ ROS-dependent apoptosis of the cells ↓ Cell-cycle in the S phase	Wu et al. (2018b)
*In vitro:* cells MCF-7, MDA-MB-231; *In vivo:* xenograft mouse model (n = 5 per group) Control: negative	Quercetin; 20, 40, 60, 80, 100 μM/24 and 48 h Manufacturer and/or supplier of the product: Invitrogen, Carlsbad, CA, United States Product name: Quercetin Minimal active concentration: 20 μM	↓ Viability of the cells ↓ Cell invasion and migration ↓ Glycolysis ↑ AKT/mTOR pathway mediated autophagy	Jia et al. (2018)
*In vitro:* cells MCF-7 and CD44^+^CD24^−^, non- CD44^+^CD24^−^ cancer stem cells subpopulations; *In vivo:* xenograft mouse model Control: negative	Quercetin; 12.5, 25, 50, 100, 200 μM/24 h or 48 h Manufacturer and/or supplier of the product: National Institute for the Control of Pharmaceutical and Biological Products, Beijing, China product name: Quercetin Minimal active concentration: 25 μM	↓ Viability of MCF-7 cells ↓ Viability of both CD44^+^CD24^−^ subpopulations ↑ Apoptosis of MCF-7 cells ↑ Number of G1 phase MCF-7 cells ↓ Tumorigenicity and metastatic ability of MCF-7 cells ↓ PI3K/AKT/mTOR-signaling	Li et al. (2018)
*In vitro:* cells MDA-MB-453 Control: negative	Kaempferol; 1–200 μM for 24 and 48 h Manufacturer and/or supplier of the product: Sigma, St. Louis, MO, United States Product name: Kaempferol Minimal active concentration: 10 μM	↓ Cell growth and proliferation ↓ Cell cycle at the G2/M phase *via* downregulation of CDK1 ↑ Apoptosis in sub-G0 phase ↑ Expression and phosphorylation of p53	Choi and Ahn (2008)
*In vitro:* cells MCF-7 Control: negative	Kaempferol; 25–100 μg/mL/48 h Manufacturer and/or supplier of the product: Sigma, St. Louis, MO, United States Product name: kaempferol Minimal active concentration: 50 μg/mL	↑ Apoptosis through the mitochondrial pathway ↑ Cellular antioxidant ability ↑ Antiproliferative activity	Liao et al. (2016)
*In vitro:* cells BT474, MDA-MB-231 Control: negative	Kaempferol; 10–4 - 10–8 M/48, 72 h Manufacturer and/or supplier of the product: Sigma-Aldrich, St. Louis, MO, United States Product name: Kaempferol Minimal active concentration: 43 μM	↓ Proliferation of the cells ↑ Number of G2 phase MDA-MB-231 cells ↑ Apoptosis mediated by caspases and DNA damage in MDA-MB-231 cells	Zhu and Xue (2019)
*In vitro:* cells MCF-7, SKBR3, MDA-MB-231, BT474; *In vivo:* xenograft mouse model (n = 6 per group) Control: negative	Kaempferol 3-arabinofuranoside (juglanin); 0–40 μM/24 and 48 h Manufacturer and/or supplier of the product: not provided Product name: Juglanin Minimal active concentration: 5 μM	↓ Proliferation of cancer cells ↑ Number of G2/M phase MCF-7 and SKBR3 cells ↑ Apoptosis of MCF-7 and SKBR3 cells ↑ JNK activation and ROS production ↓ Tumor growth in the xenograft model	Sun et al. (2017)
*In vitro:* cells MCF-7 Control: negative	Kaempferol; 10–100 μM/26 min and 24 h Manufacturer and/or supplier of the product: Sigma, St. Louis, MO, United States Product name: Kaempferol Minimal active concentration: 30 μM	↓ Glucose cellular uptake ↑ Extracellular lactate levels ↓ Cell viability, culture growth, and cell proliferation (100 μM)	Azevedo et al. (2015)
*In vitro:* cells MDA-MB-231 Control: negative	Kaempferol; 10, 20, or 40 μM/24 h Manufacturer and/or supplier of the product: Shanxi Huike Botanical Development Co., Ltd., Shanxi, China Product name: Kaempferol Minimal active concentration: 20 μM	↓ Adhesion, migration, and invasion of the cells ↓ Activity and expression of MMP-2 and MMP-9 ↓ Protein kinase Cδ and MAPK signaling	Li et al. (2015b)
*In vitro:* cells MCF-7 Control: positive and negative	Kaempferol; 25, 50 μM/24 h, 48 h, 72 h Manufacturer and/or supplier of the product: Sigma-Aldrich, St. Louis, MO, United States Product name: Kaempferol Minimal active concentration: 25 μM	↓ 17β-estradiol or triclosan-induced cell migration and invasion ↓ Protein expressions of metastasis-promoting genes induced by 17β-estradiol or triclosan	Lee et al. (2017)
*In vitro:* cells MCF-7; *In vivo:* xenograft mouse model (n = 5 per group) Control: positive and negative	Kaempferol; 50–100 μM/6 h, 4 and 6 days Manufacturer and/or supplier of the product: Abcam, Corp, Cambridge, United Kingdom Product name: Kaempferol Minimal active concentration: 50 μM	↓ 17β-estradiol or triclosan-induced cell/tumor growth ↓ Protein expressions of IGF signaling-related genes promoted by 17β-estradiol or triclosan	Kim et al. (2016b)
*In vitro:* cells MDA-MB-231 and MDA-MB-453 (triple-negative breast cancer cells); SKBR-3, MCF-7 Control: negative	Kaempferol; 10, 20, 40 μM/6 h Manufacturer and/or supplier of the product: Sigma, St. Louis, MO, United States Product name: Kaempferol Minimal active concentration: 10 μM	↓ Migration and invasion of triple-negative breast cancer cells ↓ RhoA and Rac1 signaling pathway in triple-negative breast cancer cells	Li S. et al. (2017)

Abbreviations: AKT, protein kinase B; CDK, cyclin-dependent kinase; EGFR, epidermal growth factor receptor; ER, estrogen receptor; ERK, extracellular signal-regulated kinase; HPLC-DAD, high-performance liquid chromatography with diode-array detection; HPLC-UV, high-performance liquid chromatography with ultraviolet detection; IGF, insulin-like growth factor; JNK, c-Jun N-terminal kinase; MAPK, mitogen-activated protein kinase; MMP, matrix metalloproteinase; mTOR, mammalian target of rapamycin; NF-κB, nuclear factor-kappa B; p53, tumor protein; p57, cyclin dependent kinase inhibitor 1C; PI3K, phosphatidylinositol-3, kinase; Rac1, Ras-related C3 botulinum toxin substrate 1; RhoA, Ras homolog gene family member A; ROS, reactive oxygen species; STAT3, signal transducer and activator of transcription 3; TNF, tumor necrosis factor.

Hu et al. (2015) revealed the anti-proliferative and pro-apoptotic effects of isorhamnetin in breast cancer mediated through inhibition of AKT/mTOR and MEK/ERK signaling pathways. Li et al. (2015a) discovered an inhibitory impact of isorhamnetin on the invasion of human breast carcinoma MDA-MB-231 cells by reducing the expression and activity of MMP-2 and MMP-9. This inhibition can be potentially linked to p38 MAPK and STAT3 suppression. Wu et al. (2018a) found that isorhamnetin dose-dependently inhibited the growth of human breast cancer MCF-7 cells, and exerted a strong cytotoxic effect through the reactive oxygen species (ROS)-dependent apoptosis pathway. According to Yang et al. (2023), isorhamnetin significantly reduced cell proliferation and migration and enhanced antitumor competence of doxorubicin (DOX) against resistant breast cancer cells both *in vitro* and *in vivo*, indicating its anti-breast tumor action as a DOX sensitizer.

Numerous *in vitro* studies have shown that quercetin at high concentrations exerts anti-proliferative impacts on breast cancer cells by arresting the cell cycle and inducing apoptosis (Chien et al., 2009; Chou et al., 2010; Deng et al., 2013). Conversely, lower doses of quercetin resulted in strong pro-proliferative effects (van der Woude et al., 2003; Xu et al., 2020). van der Woude et al. (2005) found that quercetin-induced stimulation of breast cancer cell proliferation was mediated by the ER. In ER^+^ (e.g., MCF-7) cells, lower concentrations of quercetin led to proliferative effects, while higher concentrations decreased cell viability. In ER^−^ (e.g., MDA-MB-231) cells, reduced cell proliferation was observed even at low doses of quercetin. According to Duo et al. (2012), quercetin at higher concentrations was able to induce apoptosis through induction of BAX with concomitant inhibition of BCL-2 in human breast cancer MCF-7 cells and also through mitochondria- and caspase-3-dependent pathways in human breast carcinoma MDA-MB-231 cells (Chien et al., 2009). Tao et al. (2015) found that quercetin strongly inhibited cell proliferation in human breast cancer cells in a time- and dose-dependent fashion, which was associated with upregulation of miR-146a expression and induction of apoptosis through activation of caspase-3 and mitochondrial-dependent pathways. Wu et al. (2018b) pointed out the antitumor effects of quercetin through the induction of ROS-dependent apoptosis in MCF-7 cells. Jia et al. (2018) revealed that quercetin suppressed breast cancer progression by inhibiting cell motility and glycolysis via the induction of autophagy mediated by the AKT/mTOR pathway. Animal studies using tumor xenografts revealed that quercetin administration led to a reduction in tumor volume and decreased the markers associated with tumor growth and metastatic properties (Tao et al., 2015; Jia et al., 2018; Li et al., 2018).

In breast cancer, kaempferol can inhibit cell growth by destroying the cell cycle and induce apoptosis through p53 phosphorylation (Choi and Ahn, 2008), mitochondria-dependent pathway (Liao et al., 2016; Zhu and Xue, 2019), ROS/c-Jun N-terminal kinase (JNK) signaling pathway (Sun et al., 2017). The primary intracellular antioxidant mechanism of kaempferol involves scavenging the ROS accumulation and maintaining the activity of antioxidant enzymes at a physiological level. According to Azevedo et al. (2015), kaempferol at high concentrations strongly inhibited glucose uptake by breast carcinoma MCF-7 cells, leading to a significant decline in cell viability and proliferative capability. Li et al. (2015b) reported that kaempferol suppressed the invasion of human breast cancer MDA-MB-231 cells by downregulating the activity and expression of MMP-9. Kaempferol was also able to inhibit triclosan-induced EMT and metastatic behavior in breast cancer MCF-7 cells (Lee et al., 2017). Zhu and Xue (2019) demonstrated that the inhibitory effects of kaempferol on cell proliferation are greater in ER^−^ (MDA-MB-231) cells compared to ER^+^ breast carcinoma (BT474) cells. According to Kim S. H. et al. (2016), kaempferol exerted anti-proliferative activity against breast cancer by suppressing triclosan- and estrogen-induced cancer progression by acting as an antagonist of ER and IGF-1R signaling in both cellular and xenograft breast cancer models. Li S. et al. (2017) revealed that a low dose of kaempferol inhibited the migration and invasion of triple-negative breast cancer (TNBC) cells by targeting the Rac1 or RhoA signaling pathway. In a mouse xenograft model, kaempferol inhibited the growth of breast cancer *in vivo* (Sun et al., 2017).

## 5 Methodological aspects and limitations of the reviewed studies

From the point of view of the interpretation and validity of the studies listed in Supplementary Table 1; Table 1, it is necessary to focus attention on several methodological aspects that should be fulfilled during pharmacological research (Heinrich et al., 2020). An important limitation of the studies analysing sea buckthorn extracts is the lack of characterization of the experimental material. Moreover, although relevant animal and cell models, negative and in several cases positive controls were used in those researches, no comparable healthy controls were available for cultured tumor cells to monitor metabolite selectivity. On the contrary, it is positive that studies showing antioxidant effects of metabolites used cell-based antioxidant assays, which, unlike chemical ones, are pharmacologically relevant. Other potential methodological risks arise from the structure and nature of flavonoids investigated. In general, polyphenols are categorized as Pan-Assay INterference compounds (PAINs or promiscuous inhibitors) and can interfere with the results of various assays. They also bind broadly to the protein targets of the assays themselves (Sheridan and Spelman, 2022). It has been revealed that polyphenols can self-associate to form colloids, which can affect their affinity for proteins. Flavonoids are more prone to aggregation than other phenolic metabolites under the conditions of the biochemical assays, with quercetin confirmed as promiscuous inhibitor (Pohjala and Tammela, 2012). Consequently, flavonoids interfere with colorimetric protein assays in a concentration- and structure-dependent manner (Singh et al., 2020) and affect other commonly used assays, such as MTT, by altering succinate dehydrogenase activity or directly interacting with MTT (Wang et al., 2010). In addition, such properties of flavonoids can be a potential source of misleading results in molecular docking analysis, therefore its findings should be verified in experimental studies. Thus, all circumstances, specifics, and risks of using individual methods should always be considered when analysing flavonoids and the failure to provide details on dealing with these methodological aspects can be considered as another limitation.

## 6 Conclusion

Botanical drugs have recently achieved remarkable success in promoting the treatment of various diseases. Sea buckthorn shows great medicinal and therapeutic potential due to its high content of bioactive metabolites with anti-proliferative, antioxidant, and anti-inflammatory activities. This review described the contemporary knowledge from *in vitro* and *in vivo* studies on the effect of sea buckthorn and its flavonoids isorhamnetin, quercetin, and kaempferol on bone and breast tissue health with an emphasis on osteoporosis and breast cancer, given their raising incidence in postmenopausal women. Conducted studies related to bone damage have demonstrated favorable impacts of all aforementioned bioactive metabolites on bone remodeling and mineralization, oxidative stress, bone microarchitecture and strength. In relation to breast cancer, sea buckthorn and its flavonoids inhibited cancer cell proliferation while inducing apoptosis, reduced tumor expansion and metastatic properties. In any case, it should be noted that several studies using extracts did not provide a sufficiently detailed definition of the study material or reports on the phytochemical analysis of the extracts as recommended by the best practice guidelines, indicating limitations and lower reliability of these studies. In addition, the known interference of flavonoids with commonly used assays (such as protein or MTT assays) should be always considered and may be another source of limitation. On the contrary, all these investigations used standard research models or cell lines and were published in peer-reviewed journals. By evaluating the available studies that analysed extracts and flavonoids mentioned in our manuscript, we can state that our hypothesis was confirmed, as all bioactive metabolites improved the impaired health status of both bone and breast tissues. In addition, some research has investigated the role of sea buckthorn extracts in reducing chemotherapy- and radiotherapy-related side effects, suggesting their potential benefits to improve overall treatment outcomes. Further *in vitro* studies and animal model studies that provide enough detailed information on the investigated material are needed, as well as clinical trials involving osteoporotic/non-osteoporotic and breast cancer/non-breast cancer patients, which may provide the key findings for identifying more effective therapies against bone and breast tissue damage. In this regard, the appropriate selection of the optimal dose and type of bioactive agent for inducing protective effects on bone and breast tissues in humans requires careful consideration and further validation in clinical trials.
