# Supplementary material for: Sea buckthorn and its flavonoids isorhamnetin, quercetin, and kaempferol favorably influence bone and breast tissue health
Source: Front Pharmacol. 2024 Oct 9;15:1462823. doi: 10.3389/fphar.2024.1462823 (PMC11497132; doi:10.3389/fphar.2024.1462823)
Supplement: Supplementary file 1 [file Table1.docx]

**Supplementary Table 1.** *In vitro* and *in vivo* studies reflecting the potential of sea buckthorn and its flavonoids isorhamnetin, quercetin, and kaempferol against bone damage.

| **Research Model** | **Applied Treatment and Metabolite Description** | **Obtained Results** | **References** |
| --- | --- | --- | --- |
| *In vivo:* rats  Sprague–Dawley, OVX (n=6 per group)  Control: positive and negative  (n=4 and 6 per group) | sea buckhorn;  10 ml/kg twice daily / 6 weeks  Origin: purchased from a University center  Minimal active concentration: 10/ml/kg  No data about herbal parts used, authentication of the plant material, locality and date of harvesting, deposition of voucher specimen, details about plant material processing. | ↑ Estrogen, P1NP, CTX  ↑ BMD, BMC  ↑ BV/TV, Tb.Th  ↓ Tb.N, Tb.Sp | Yuan et al. (2022) |
| *In vivo:* mice  ICR, OVX (n=10 per group)  Control: positive and negative  (n=6 and 10 per group) | extracts of sea buckhorn fruits and their fractions;  50 and 150 mg/kg once a day / 12 weeks  Herbal parts: freeze-dried fruits  Authentication: one of the authors  Origin: purchased from a commercial company  Voucher specimen deposited: in an institutional herbarium  Minimal active dose: 50 mg/kg  Extraction details: solvent (70% ethanol, distilled water, hexane, chloroform, ethyl acetate, and n-butanol), type (liquid), fractionated by preparative HPLC  No data about locality and date of harvesting, extract characterization. | ↑ BMD  ↓ Bone marrow fat  ↓ Cartilage damage and disruption of trabecular bone structure  ↑ ALP, OPN, RUNX2, OSX | Park et al. (2022) |
| *In vitro:* cells  C3H10T1/2  Control: positive and negative | extracts of sea buckhorn fruits and their fractions;  50 μg/ml / 9 days  Herbal parts: freeze-dried fruits  Authentication: one of the authors  Origin: purchased from a commercial company  Voucher specimen deposited: in an institutional herbarium  Minimal active dose: 50 μg/ml  Extraction details: solvent (70% ethanol, distilled water, hexane, chloroform, ethyl acetate, and n-butanol), type (liquid), fractionated by preparative HPLC  No data about locality and date of harvesting, extract characterization. | ↑ ALP, OPN, RUNX2, OSX | Park et al. (2022) |
| *In vitro:* cells  C3H10T1/2  Control: positive and negative | extracts of sea buckhorn fruits and their fractions;  10 μg/ml / 9 days  Herbal parts: freeze-dried fruits  Authentication: one of the authors  Origin: purchased from a commercial company  Voucher specimen deposited: in an institutional herbarium  Minimal active dose: 10 μg/ml  Extraction details: solvent (70% ethanol, distilled water, hexane, chloroform, ethyl acetate, and n-butanol), type (liquid), fractionated by preparative HPLC  No data about locality and date of harvesting, extract characterization. | ↑ ALP, OPN, RUNX2, OSX | Lee et al. (2023) |
| *In vitro:* mouse bone marrow cells  Control: positive and negative | isorhamnetin;  10^−8^ - 10^−6^M / 7 days  Manufacturer and/or supplier of the product: Sigma-Aldrich, St. Louis, MO, USA  Product name: Isorhamnetin  Minimal active concentration: 10^−8^M | ↓ PTH-induced osteoclast-like cell formation | Yamaguchi et al. (2007) |
| *In vitro:* cells of  rat femoral-diaphyseal and metaphyseal tissues  Control: negative | isorhamnetin;  10^−6^M / 48 h  Manufacturer and/or supplier of the product: Sigma-Aldrich, St. Louis, MO, USA  Product name: Isorhamnetin  Minimal active concentration: 10^−6^M | ↑ Calcium content decreased by PTH | Yamaguchi et al. (2007) |
| *In vitro:* mice primary bone marrow macrophages  Control: positive and negative | isorhamnetin;  3.125, 6.25, 12.5, 25, 50, 100 and 150 μM / 24 h  Manufacturer and/or supplier of the product: Herbpurify, Chengdu, China  Product name: Isorhamnetin  Minimal active concentration: 3.125 μM | ↓ RANKL‐induced ROS generation  ↓ Osteoclast formation  ↓ RANKL‐induced activation of MAPK, NF‐κB, and AKT signaling | Zhou et al. (2019) |
| *In vitro:* mouse bone marrow cells and  RAW264.7  Control: positive and negative | quercetin;  1 - 5 μM  Manufacturer and/or supplier of the product: Wako Pure Chemical  Industries, Ltd., Osaka, Japan  Product name: Quercetin  Minimal active concentration: 1 μM | ↓ Differentiation of osteoclast progenitor cells  ↓ TRAP activity | Woo et al. (2004) |
| *In vitro:* cells  MC3T3 and RAW264.7  Control: positive and negative | quercetin;  0.1, 1, 10, 25, 50 µM / 6 or 21 days  Manufacturer and/or supplier of the product: Sigma-Aldrich, St. Louis, MO, USA  Product name: Quercetin  Minimal active concentration: 0.1 µM | ↓ RANKL-induced osteoclastogenesis  ↓ Basal and TNFα-induced NF-κB activity  ↓ TGFβ and BMP-2-induced SMAD activation | Yamaguchi and Weitzmann (2011) |
| *In vitro:* cells  RAW264.7 7 Control: positive and negative | quercetin;  1, 2, or 5 μM / 2 days  Manufacturer and/or supplier of the product: National Institutes for Food and Drug Control, Beijing, China  Product name: Quercetin  Minimal active concentration: 2 μM | ↓ RANKL-induced osteoclastogenesis  ↑ *STC1* gene expression  ↓ ROS level  ↓ IL-6, TNFα, CTSK, NFATc1 expression | Niu et al. (2020) |
| *In vitro:* RANKL-treated RAW264.7 cells  Control: negative | quercetin incorporated into 3D porous calcium deficient hydroxyapatite scaffolds;  1, 10, 100, 200 μM / 1, 3, 5 days  Manufacturer and/or supplier of the product: Wako Pure Chemical  Industries, Ltd., Osaka, Japan  Product name: Quercetin  Minimal active concentration: 200 μM | ↓ Cell proliferation  ↓ TRAP activity | Tripathi et al. (2015) |
| *In vitro:* cells  RAW264.7  Control: positive and negative | quercetin;  1, 5, 10 μM / 4 or 5 days  Manufacturer and/or supplier of the product: Sigma-Aldrich, St. Louis, MO, USA  Product name: Quercetin  Minimal active concentration: 1 μM | ↓ RANKL-induced osteoclast formation  ↓ RANKL-induced NFATc1, CTR, CTSK, MMP-9 expression | Tsuji et al. (2009) |
| *In vitro:* cells  MC3T3-E1  Control: negative | quercetin incorporated into 3D porous calcium deficient hydroxyapatite scaffolds;  1, 10, 100, 200 μM / 1, 3, 5 days  Manufacturer and/or supplier of the product Wako Pure Chemical  Industries, Ltd., Osaka, Japan  Product name: Quercetin  Minimal active concentration: 200 μM | ↑ Cell proliferation  ↑ ALP activity  ↑ RUNX2, collagen I, BSP, OC gene expression  ↑ Calcium mineralization | Tripathi et al. (2015) |
| *In vitro:* cells  ROS 17/2.8  Control: negative | quercetin;  5 µM / 12 h  Manufacturer and/or supplier of the product: Wako Pure Chemical  Industries, Ltd., Osaka, Japan  Product name: Quercetin  Minimal active concentration: 5 μM | ↑ BSP, RUNX2 expression | Kim et al. (2007) |
| *In vitro:* cells  MG-63  Control: negative | quercetin;  50 μM / 24 or 48 h  Manufacturer and/or supplier of the product: Sigma-Aldrich, St. Louis, MO, USA  Product name: Quercetin  Minimal active concentration: 50 μM | ↑ ALP activity  ↑ ERK pathway | Prouillet et al. (2004) |
| *In vitro:* BMSCs isolated from Sprague Dawley rats  Control: positive and negative | quercetin;  1 μM / 24 h  Manufacturer and/or supplier of the product: Sigma-Aldrich, St. Louis, MO, USA  Product name: Quercetin  Minimal active concentration: 1 μM | ↑ Cell viability  ↑ Calcium nodule formation  ↓ TNFα-induced activation of NF-κB and degradation of β-catenin  ↓ Inhibitory effect of TNFα on RUNX2 and OSX expression | Yuan et al. (2018) |
| *In vitro:* cells  BMSCs  Control: positive and negative | quercetin;  0 to 100 μM / 3, 7, 14 days  Manufacturer and/or supplier of the product: Sigma-Aldrich, St. Louis, MO, USA  Product name: Quercetin  Minimal active concentration: 25 μM | ↑ Wnt/β-catenin signaling  ↓ NF-κB signaling  ↑ TNFα-impaired osteogenesis (ALP activity)  ↑ RUNX2, ALP, OCN and BMP-2 expression | Feng et al. (2023) |
| *In vivo:* rats  Sprague Dawley, OVX  (n=10 per group)  Control: positive and negative  (n=10 per group) | quercetin;  50 mg/kg/day / 8 weeks  Manufacturer and/or supplier of the product: Sigma-Aldrich, St. Louis, MO, USA  Product name: Quercetin  Minimal active dose: 50 mg/kg | ↑ BMD  ↑ Tb.N, Tb.Th  ↑ Radialis elasticity, elastic load and maximum load (biomechanics-related indicators) | Yuan et al. (2018) |
| *In vivo:* rats  Y59 strain with retinoic acid-induced osteoporosis and healthy rats  (n=7–10 per group)  Control: positive and negative  (n=7–10 per group) | quercetin;  100 mg/kg/day / 14 days  Manufacturer and/or supplier of the product: Aldrich Ch. Co. Inc.  Milwauke WI, USA  Product name: Quercetin  Minimal active dose: 100 mg/kg | ↑ BMD, BMC, OC, Ca, P  ↓ ALP  ↑ Cortical bone thickness | Oršolić et al. (2018) |
| *In vivo:* mice  C57BL/6, ORX  (n=9 per group)  Control: positive and negative  (n=9 per group) | quercetin;  75 and 150 mg/kg/day / 8 weeks  Manufacturer and/or supplier of the product: not included  Product name: Quercetin  Minimal active dose: 75 mg/kg | ↑ Trabecular BMD, BV/TV, Tb.N, Tb.Th  ↓ Tb.Sp  ↓ Bone resorption  ↑ Maximum load and deflection, fracture energy, stiffness | Sun et al. (2022) |
| *In vivo:* mice  C57BL/6, OVX  (n=6 per group)  Control: positive and negative  (n=6 per group) | quercetin;  50 mg/kg twice per week / 4 weeks  Manufacturer and/or supplier of the product: Sigma-Aldrich, St. Louis, MO, USA  Product name: Quercetin  Minimal active dose: 50 mg/kg | ↑ BMD, BV/TV, Tb.N, Tb.Th  ↓ Tb.Sp  ↑ Bone growth, osteogenesis (mineral apposition), number of osteoblast/bone surface | Feng et al. (2023) |
| *In vivo:* mice  C57BL/6J, OVX  (n=6-7 per group)  Control: positive and negative  (n=6-7 per group) | quercetin;  0.25% or 2.5% in diet / 4 weeks  Manufacturer and/or supplier of the product: Sigma-Aldrich, St. Louis, MO, USA  Product name: Quercetin-dihydrate  Minimal active dose: 0.25% | ↑ BMD, trabecular BV/TV, Ct.Ar, Ct.Th  ↑ Osteoid surface per bone surface | Tsuji et al. (2009) |
| *In vivo:* mice  C57BL/6J with hindlimb suspension  (n=6 per group)  Control: positive and negative  (n=6 per group) | quercetin;  25, 50, 100 mg/kg/day / 4 weeks  Manufacturer and/or supplier of the product: National Institutes for Food and Drug Control, Beijing, China  Product name: Quercetin  Minimal active dose: 50 mg/kg | ↑ Trabecular BV/TV, Tb.N  ↑ Cortical volume and thickness  ↑ Stiffness in bone midpoint  ↓ CTX, TRACP5b  ↑ P1NP  ↓ Number of osteoclasts | Niu et al. (2020) |
| *In vivo:* rats  Wistar-Albino  (n=8 per group)  Control: positive and negative  (n=8 per group) | quercetin;  100 mg/kg/day / 2-6 weeks  Manufacturer and/or supplier of the product: Sigma-Aldrich, St. Louis, MO, USA  Product name: Quercetin  Minimal active dose: 100 mg/kg | ↑ Fracture strength in a healing bone  ↓ Acid phosphatase, total oxidant status  ↑ Total antioxidant status | Yurteri et al. (2023) |
| *In vitro:* mouse bone marrow cells  Control: positive and negative | quercetin;  10^−8^ - 10^−6^M / 7 days  Manufacturer and/or supplier of the product: Sigma, St. Louis, MO, USA  Product name: Quercetin  Minimal active concentration: 10^−7^ M | ↓ PTH-induced osteoclast-like cell formation | Yamaguchi et al. (2007) |
| *In vitro:* cells  MC3T3-E1, RAW264.7  Control: positive and negative | kaempferol;  10, 20 μM / 1 or 18 h  Manufacturer and/or supplier of the product: Indofine Chemical Company Inc., Belle Mead, NJ, USA  Product name: Kaempferol  Minimal active concentration: 10 μM | ↓ TNFα-induced NF-κB activation  ↓ RANKL-induced osteoclastogenesis  ↓ RANKL-induced c-Fos expression | Pang et al. (2006) |
| *In vitro:* mouse bone marrow cells  Control: positive and negative | kaempferol;  50, 100 or 200 μM / 3 days  Manufacturer and/or supplier of the product: Sigma-Aldrich, St. Louis, MO, USA  Product name: Kaempferol  Minimal active concentration: 100 μM | ↓ RANKL-mediated or IL-1β-stimulated osteoclastogenesis  ↓ RANKL or IL-1β –induced c-Fos and NFATc1 expression  ↓ RANKL-mediated or IL-1β-stimulated activation of intracellular MAPKs | Lee et al. (2014) |
| *In vitro:* cells  RAW 264.7  Control: positive and negative | kaempferol;  5, 10, 25, 50, 75, and 100 μM / 24 h  Manufacturer and/or supplier of the product: Sigma, St. Louis, MO, USA  Product name: Kaempferol  Minimal active concentration: 25 μM | ↓ RANKL-stimulated osteoclast-like cell formation  ↓ RANKL-induced ERK and JNK activation | Kim et al. (2018) |
| *In vitro:* cells  MC3T3-E1  Control: negative | kaempferol;  1, 2.5, 5, 10, 50 and 100 μM / 24–72 h and 3 to 7 days  Manufacturer and/or supplier of the product: Sigma, St. Louis, MO, USA  Product name: Kaempferol  Minimal active concentration: 10 μM | ↑ ALP activity (up to 10 μM)  ↑ RUNX2, OSX, BMP-2 and collagen I expression  ↑ Autophagy | Kim et al. (2016a) |
| *In vitro:* cells  MC3T3-E1 treated with dexamethasone  Control: positive and negative | kaempferol;  5, 10, and 25 μM / 3, 5 days and 2 weeks  Manufacturer and/or supplier of the product: not provided  Minimal active concentration: 5 μM | ↑ Cell proliferation (up to 10 μM)  ↓ Cell cycle arrest, apoptosis  ↑ ALP activity, mineralization  ↑ RUNX2 and OSX expression  ↑ p38-MAPK and JNK MAPK pathways | Xie et al. (2021) |
| *In vitro:* rat BMSCs  Control: positive and negative | kaempferol;  10 μM / 24 h  Manufacturer and/or supplier of the product: Sigma-Aldrich, St. Louis, MO, USA  Product name: Kaempferol  Minimal active concentration: 10 μM | ↑ Calcification, ALP activity, SOX2 level  ↑ PI3K/AKT/mTOR signaling  ↓ miR-124-3p level | Gan et al. (2022) |
| *In vitro:* cells  MC3T3-E1  Control: negative | kaempferol;  5 and 7.5 μM / 24 and 48 h  Manufacturer and/or supplier of the product: MedChemExpress  Product name: Kaempferol  Minimal active concentration: 5 μM | ↑ *AKT1* gene expression  ↓ *MMP-9* gene expression | Dong et al. (2024) |
| *In vitro:* cells  MG-63  Control: negative | kaempferol;  10^−6^ M / 24 h  Manufacturer and/or supplier of the product: Tauto Biotech, Shanghai, China  Product name: Kaempferol  Minimal active concentration: 10^−6^ M | ↑ Transcriptional activity of ERE reporter | Tang et al. (2008) |
| *In vitro:* rat primary calvarial osteoblasts and bone marrow cells  Control: negative | kaempferol;  0.2, 1 and 5 μM / 11 and 21 days  Manufacturer and/or supplier of the product: Sigma–Aldrich, St. Louis, MO, USA  Product name: Kaempferol  Minimal active concentration: 0.2 μM | ↑ Mineralization, bone forming by osteoblasts  ↓ Differentiation of bone marrow cells to adipocytes | Trivedi et al. (2008) |
| *In vivo:* rats  Sprague Dawley, OVX  (n=10 per group)  Control: positive and negative (n=10 per group) | kaempferol;  5 mg/kg/day / 10 weeks  Manufacturer and/or supplier of the product: Sigma–Aldrich, St. Louis, MO, USA  Product name: Kaempferol  Minimal active dose: 5 mg/kg | ↑ BMD in trabecular regions  ↑ Vertebral bone strength | Trivedi et al. (2008) |
| *In vivo:* rats  Wistar, OVX  (n=8 per group)  Control: positive and negative (n=8 per group) | kaempferol;  5 mg/kg/day / 8 weeks  Manufacturer and/or supplier of the product: not provided  Product name: Kaempferol  Minimal active dose: 5 mg/kg | ↑ BV/TV and trabecular bone perimeter  ↑ Young's modulus  ↓ Bone turnover | Nowak et al. (2017) |
| *In vivo:* rats  Sprague Dawley, OVX  (n=6 per group)  Control: positive and negative (n=6 per group) | kaempferol;  5 mg/kg/day / 12 weeks  Manufacturer and/or supplier of the product: National Institute for the Control of Pharmaceutical and Biological Products, Beijing, China  Product name: Kaempferol  Minimal active dose: 5 mg/kg | ↑ BMD, BV/TV  ↑ RUNX2, OSX, CXCL12 expression | Liu et al. (2021) |
| *In vitro:* rat BMSCs  Control: negative | kaempferol;  1 μM  Manufacturer and/or supplier of the product: National Institute for the Control of Pharmaceutical and Biological Products, Beijing, China  Product name: Kaempferol  Minimal active concentration: 1 μM | ↑ Bone mineralization ability  ↑ALP activity  ↑ RUNX2, OSX, and CXCL12 expression  ↓ miR-10a-3p | Liu et al. (2021) |

Abbreviations: AKT - protein kinase B; ALP - alkaline phosphatase; BMC - bone mineral content; BMD - bone mineral density; BMP-2 - bone morphogenetic protein 2; BMSCs - bone marrow mesenchymal stem cells; BSP - bone sialoprotein; BV/TV - relative bone volume; Ca – calcium; Ct.Ar - cortical area; Ct.Th - cortical thickness; CTSK - cathepsin K; CTR - calcitonin receptor; CTX - C-terminal telopeptide of type I collagen; CXCL12 - C-X-C motif ligand 12; ERE - estrogen response element; ERK - extracellular regulated kinase; HPLC - high performance liquid chromatography; IL-1β - interleukin 1 beta; IL-6 - interleukin-6; JNK - c-Jun N-terminal kinase; MAPK(s) - mitogen‐activated protein kinase(s); MMP-9 - matrix metalloprotease 9; mTOR - mammalian target of rapamycin; NF-κB - nuclear factor-kappa B; NFATc1 - nuclear factor of activated T cells c1; OC - osteocalcin; OPN – osteopontin; ORX – orchiectomy; OSX – osterix; OVX - ovariectomized; P – phosphorus; P1NP – pro-collagen type I amino-terminal propeptide; PI3K - phosphoinositide 3-kinase; PTH - parathyroid hormone; RANKL - receptor activator of nuclear factor kappa-Β ligand; ROS - reactive oxygen species; RUNX2 - runt-related transcription factor-2; SMAD - small mother against decapentaplegic; SOX2 - SRY-Box Transcription Factor 2; STC1 - stanniocalcin 1; Tb.N - trabecular number; Tb.Sp - trabecular separation; Tb.Th - trabecular thickness; TGFβ - transforming growth factor beta; TNFα - tumor necrosis factor alpha; TRACP5b - tartrate-resistant acid phosphatase 5b; TRAP - tartrate-resistant acid phosphatase.

**References**

Dong, Q., Ren, G., Li, Y., and Hao, D. (2024). Network pharmacology analysis and experimental validation to explore the mechanism of kaempferol in the treatment of osteoporosis. *Sci Rep* 14, 7088. doi: 10.1038/s41598-024-57796-3

Feng, L., Yang, Z., Hou, N., Wang, M., Lu, X., Li, Y., et al. (2023). Long Non-Coding RNA Malat1 Increases the Rescuing Effect of Quercetin on TNFα-Impaired Bone Marrow Stem Cell Osteogenesis and Ovariectomy-Induced Osteoporosis. *International Journal of Molecular Sciences* 24, 5965. doi: 10.3390/ijms24065965

Gan, L., Leng, Y., Min, J., Luo, X.-M., Wang, F., and Zhao, J. (2022). Kaempferol promotes the osteogenesis in rBMSCs via mediation of SOX2/miR-124-3p/PI3K/Akt/mTOR axis. *European Journal of Pharmacology* 927, 174954. doi: 10.1016/j.ejphar.2022.174954

Kim, C.-J., Shin, S.-H., Kim, B.-J., Kim, C.-H., Kim, J.-H., Kang, H.-M., et al. (2018). The Effects of Kaempferol-Inhibited Autophagy on Osteoclast Formation. *Int J Mol Sci* 19, 125. doi: 10.3390/ijms19010125

Kim, D.-S., Takai, H., Arai, M., Araki, S., Mezawa, M., Kawai, Y., et al. (2007). Effects of quercetin and quercetin 3-glucuronide on the expression of bone sialoprotein gene. *J Cell Biochem* 101, 790–800. doi: 10.1002/jcb.21233

Kim, I.-R., Kim, S.-E., Baek, H.-S., Kim, B.-J., Kim, C.-H., Chung, I.-K., et al. (2016a). The role of kaempferol-induced autophagy on differentiation and mineralization of osteoblastic MC3T3-E1 cells. *BMC Complementary and Alternative Medicine* 16, 333. doi: 10.1186/s12906-016-1320-9

Lee, D., Park, K., Hong, J.-H., Kim, S., Park, K.-M., and Kim, K. H. (2023). Anti-osteoporosis effects of triterpenoids from the fruit of sea buckthorn (Hippophae rhamnoides) through the promotion of osteoblast differentiation in mesenchymal stem cells, C3H10T1/2. *Archives of Pharmacal Research* 46. doi: 10.1007/s12272-023-01468-9

Lee, W.-S., Lee, E.-G., Sung, M.-S., and Yoo, W.-H. (2014). Kaempferol Inhibits IL-1β-Stimulated, RANKL-mediated Osteoclastogenesis via Downregulation of MAPKs, c-Fos, and NFATc1. *Inflammation* 37, 1221–1230. doi: 10.1007/s10753-014-9849-6

Liu, H., Yi, X., Tu, S., Cheng, C., and Luo, J. (2021). Kaempferol promotes BMSC osteogenic differentiation and improves osteoporosis by downregulating miR-10a-3p and upregulating CXCL12. *Mol Cell Endocrinol* 520, 111074. doi: 10.1016/j.mce.2020.111074

Niu, Y., Yang, Y., Xiao, X., Sun, Y., Zhou, Y., Zhang, Y., et al. (2020). Quercetin prevents bone loss in hindlimb suspension mice via stanniocalcin 1-mediated inhibition of osteoclastogenesis. *Acta Pharmacol Sin* 41, 1476–1486. doi: 10.1038/s41401-020-00509-z

Nowak, B., Matuszewska, A., Nikodem, A., Filipiak, J., Landwójtowicz, M., Sadanowicz, E., et al. (2017). Oral administration of kaempferol inhibits bone loss in rat model of ovariectomy-induced osteopenia. *Pharmacol Rep* 69, 1113–1119. doi: 10.1016/j.pharep.2017.05.002

Oršolić, N., Jeleč, Ž., Nemrava, J., Balta, V., Gregorović, G., and Jeleč, D. (2018). Effect of Quercetin on Bone Mineral Status and Markers of Bone Turnover in Retinoic Acid-Induced Osteoporosis. *Pol. J. Food Nutr. Sci.* 68, 149–162. doi: 10.1515/pjfns-2017-0023

Pang, J. L., Ricupero, D. A., Huang, S., Fatma, N., Singh, D. P., Romero, J. R., et al. (2006). Differential activity of kaempferol and quercetin in attenuating tumor necrosis factor receptor family signaling in bone cells. *Biochemical Pharmacology* 71, 818–826. doi: 10.1016/j.bcp.2005.12.023

Park, K. H., Hong, J.-H., Kim, S.-H., Kim, J.-C., Kim, K. H., and Park, K.-M. (2022). Anti-Osteoporosis Effects of the Fruit of Sea Buckthorn (Hippophae rhamnoides) through Promotion of Osteogenic Differentiation in Ovariectomized Mice. *Nutrients* 14, 3604. doi: 10.3390/nu14173604

Prouillet, C., Mazière, J.-C., Mazière, C., Wattel, A., Brazier, M., and Kamel, S. (2004). Stimulatory effect of naturally occurring flavonols quercetin and kaempferol on alkaline phosphatase activity in MG-63 human osteoblasts through ERK and estrogen receptor pathway. *Biochemical Pharmacology* 67, 1307–1313. doi: 10.1016/j.bcp.2003.11.009

Sun, J., Pan, Y., Li, X., Wang, L., Liu, M., Tu, P., et al. (2022). Quercetin Attenuates Osteoporosis in Orchiectomy Mice by Regulating Glucose and Lipid Metabolism via the GPRC6A/AMPK/mTOR Signaling Pathway. *Front. Endocrinol.* 13. doi: 10.3389/fendo.2022.849544

Tang, X., Zhu, X., Liu, S., Nicholson, R. C., and Ni, X. (2008). Phytoestrogens induce differential estrogen receptor β-mediated responses in transfected MG-63 cells. *Endocr* 34, 29–35. doi: 10.1007/s12020-008-9099-1

Tripathi, G., Raja, N., and Yun, H. S. (2015). Effect of direct loading of phytoestrogens into the calcium phosphate scaffold on osteoporotic bone tissue regeneration. *J. Mater. Chem. B* 3, 8694–8703. doi: 10.1039/C5TB01574J

Trivedi, R., Kumar, S., Kumar, A., Siddiqui, J. A., Swarnkar, G., Gupta, V., et al. (2008). Kaempferol has osteogenic effect in ovariectomized adult Sprague-Dawley rats. *Mol Cell Endocrinol* 289, 85–93. doi: 10.1016/j.mce.2008.02.027

Tsuji, M., Yamamoto, H., Sato, T., Mizuha, Y., Kawai, Y., Taketani, Y., et al. (2009). Dietary quercetin inhibits bone loss without effect on the uterus in ovariectomized mice. *J Bone Miner Metab* 27, 673–681. doi: 10.1007/s00774-009-0088-0

Woo, J.-T., Nakagawa, H., Notoya, M., Yonezawa, T., Udagawa, N., Lee, I.-S., et al. (2004). Quercetin suppresses bone resorption by inhibiting the differentiation and activation of osteoclasts. *Biol Pharm Bull* 27, 504–509. doi: 10.1248/bpb.27.504

Xie, B., Zeng, Z., Liao, S., Zhou, C., Wu, L., and Xu, D. (2021). Kaempferol Ameliorates the Inhibitory Activity of Dexamethasone in the Osteogenesis of MC3T3-E1 Cells by JNK and p38-MAPK Pathways. *Front. Pharmacol.* 12. doi: 10.3389/fphar.2021.739326

Yamaguchi, M., Hamamoto, R., Uchiyama, S., and Ishiyama, K. (2007). Effects of flavonoid on calcium content in femoral tissue culture and parathyroid hormone-stimulated osteoclastogenesis in bone marrow culture in vitro. *Mol Cell Biochem* 303, 83–88. doi: 10.1007/s11010-007-9458-x

Yamaguchi, M., and Weitzmann, M. N. (2011). Quercetin, a potent suppressor of NF-κB and Smad activation in osteoblasts. *Int J Mol Med* 28, 521–525. doi: 10.3892/ijmm.2011.749

Yuan, Y.-F., Wang, S., Zhou, H., Tang, B.-B., Liu, Y., Huang, H., et al. (2022). Exploratory study of sea buckthorn enhancing QiangGuYin efficacy by inhibiting CKIP-1 and Notum activating the Wnt/β-catenin signaling pathway and analysis of active ingredients by molecular docking. *Front. Pharmacol.* 13. doi: 10.3389/fphar.2022.994995

Yuan, Z., Min, J., Zhao, Y., Cheng, Q., Wang, K., Lin, S., et al. (2018). Quercetin rescued TNF-alpha-induced impairments in bone marrow-derived mesenchymal stem cell osteogenesis and improved osteoporosis in rats. *Am J Transl Res* 10, 4313–4321. Available at: https://www.ncbi.nlm.nih.gov/pmc/articles/PMC6325508/ (Accessed June 20, 2024).

Yurteri, A., Yildirim, A., Çelik, Z. E., Vatansev, H., and Durmaz, M. S. (2023). The effect of quercetin on bone healing in an experimental rat model. *Jt Dis Relat Surg* 34, 365–373. doi: 10.52312/jdrs.2023.870

Zhou, F., Mei, J., Yuan, K., Han, X., Qiao, H., and Tang, T. (2019). Isorhamnetin attenuates osteoarthritis by inhibiting osteoclastogenesis and protecting chondrocytes through modulating reactive oxygen species homeostasis. *J Cell Mol Med* 23, 4395–4407. doi: 10.1111/jcmm.14333
